# Bile Acid Alters Male Mouse Fertility in Metabolic Syndrome Context

**DOI:** 10.1371/journal.pone.0139946

**Published:** 2015-10-06

**Authors:** Aurélie Vega, Emmanuelle Martinot, Marine Baptissart, Angélique De Haze, Frederic Vaz, Wim Kulik, Christelle Damon-Soubeyrand, Silvère Baron, Françoise Caira, David H. Volle

**Affiliations:** 1 INSERM U 1103, Génétique Reproduction et Développement (GReD), F–63178 Aubière, France; 2 Université Clermont Auvergne, GReD, F–63178 Aubière, France; 3 CNRS, UMR 6293, GReD, F–63178 Aubière, France; 4 Centre de Recherche en Nutrition Humaine d’Auvergne, F–63000 Clermont-Ferrand, France; 5 Academic Medical Center, University of Amsterdam, Lab. Genetic Metabolic Diseases, F0-224, P.O. Box 22700, 1100 DE Amsterdam, The Netherlands; IRCCS Istituto Oncologico Giovanni Paolo II, ITALY

## Abstract

Bile acids have recently been demonstrated as molecules with endocrine activities controlling several physiological functions such as immunity and glucose homeostases. They act mainly through two receptors, the nuclear receptor Farnesol-X-Receptor alpha (FXRα) and the G-protein coupled receptor (TGR5). These recent studies have led to the idea that molecules derived from bile acids (BAs) and targeting their receptors must be good targets for treatment of metabolic diseases such as obesity or diabetes. Thus it might be important to decipher the potential long term impact of such treatment on different physiological functions. Indeed, BAs have recently been demonstrated to alter male fertility. Here we demonstrate that in mice with overweight induced by high fat diet, BA exposure leads to increased rate of male infertility. This is associated with the altered germ cell proliferation, default of testicular endocrine function and abnormalities in cell-cell interaction within the seminiferous epithelium. Even if the identification of the exact molecular mechanisms will need more studies, the present results suggest that both FXRα and TGR5 might be involved. We believed that this work is of particular interest regarding the potential consequences on future approaches for the treatment of metabolic diseases.

## Introduction

Metabolic syndrome (MetS) has been linked with several abnormalities including overweight, dyslipidemia, hypertension and impaired glucose metabolism [1]. The numerous deleterious effects of MetS are being investigated throughout the medical community as MetS may affect many aspects of human physiology due to its systemic nature.

It has been proposed since 10 years that derivatives of bile acids (BAs) could be interesting molecules for the treatment of diseases of MetS such as diabetes or obesity. Indeed, BAs are being appreciated as complex metabolic integrators and signaling factors [2]. Through activation of diverse signaling pathways, BAs regulate their own synthesis, enterohepatic recirculation, as well as triglyceride, cholesterol, energy and glucose homeostases. BAs act mainly through two specific receptors, the nuclear receptor Farnesol-X-Receptor alpha (FXRα) and the G-protein coupled receptor TGR5 [3]. In order to develop promising novel drug targets to treat common hepatic and metabolic diseases, a lot of work has been done to better define the respective involvement of both receptors in different physiological pathways

This is a challenge as synthetic agonist for either FXRαor TGR5 could have either beneficial or deleterious impacts. Indeed, if FXRα agonists seem to be good candidate in treatment of diabetes [4], their utilization for the treatment of obesity can worsen the pathology [4]. Thus clear establishment of the involvement of each BA signaling pathways remain to be defined before using them as therapeutical drugs. Moreover, activation of such important signaling pathways could have some secondary effects on health.

In that line, our recent findings demonstrate the impact of BA on male fertility and testicular physiology. Indeed, BAs were shown to impact testicular physiology either via TGR5 or FXRα. TGR5 mainly acts in germ cell lineage. Its activation by BA represses network of cell-cell interactions through the downregulation of N-Cadherin as well as Cx43 expression through regulation of the transcriptional repressor, T-box transcription factor 2 gene [5]. This leads to germ cell sloughing and rupture of the blood-testis barrier and then apoptosis of spermatids. In parallel, FXRα has been demonstrated in several studies to control the testicular endocrine function supported by the Leydig cells *in vivo* [6]. Short term exposure to FXRα agonist (GW4064) represses testosterone synthesis. At the molecular level, FXRαactivation stimulates the expression of the small heterodimer partner (SHP) which in turn inhibits the expression of steroidogenic genes, on the one hand by inhibiting the expression of the nuclear receptors steroidogenic factor–1 (SF–1) and liver receptor homolog–1 (LRH–1), and on the other hand by directly repressing the transcriptional activity of LRH–1.

Regarding the links between BA signaling pathways and male testicular physiology and subsequent fertility disorders, we wondered what could be the consequences of a long term exposure to molecules that activate BA signaling pathways during the treatment of MetS diseases such as obesity. We thus aimed to define the equilibrium between potential positive or negative impacts of “BA treatment”. We asked whether the BA-treatment will improve general heath of the mice and then fertility, or if BA will mainly have deleterious effects on fertility as we have demonstrated recently [5]. For that purpose, we decided to induce overweight in mice with high fat-diet (HFD) and then “treat” them with BAs to reverse the overweight as defined by Watanabe *et al*. [7].

Here we demonstrate that in mice fed a high fat diet, BA exposure leads to increased rate of male infertility. This is associated with alterations of testicular physiology.

## Materials and Methods

### Ethics Statement

This study was conducted in accordance with the current regulations and standards approved by the Animal Care Committee (CEMEA Auvergne; protocol CE 08–12).

### Animals

C57Bl/6J were purchased from Charles River Laboratories (L'Arbresle, France). Mice were housed in temperature-controlled rooms with 12 hours light/dark cycles. Mice had *ad libitum* access to food and water. Five-week-old mice were exposed to D04-diet or 235HF diet (Control) for 3 months. Half of the group fed HFD were maintained either on 235HF diet or the 235HF supplemented with 0.5% cholic acid (HF-CA-diet) (SAFE, Augy, France) for 2 or 4 months to visualize the impact of BA exposure on overweight.

### Fertility assement

Fifteen days before the sacrifice each male is put into reproduction during the day, without food (avoiding contamination of the females), with two unexposed C57Bl6J females (Charles River) (3 to 4 males per group per experiment). Breeding was daily monitored for the presence of a vaginal plug to determine whether mating occurred. Before the night, males were put back with specific diet (regarding the group). After 19–20 d, efficacy of mating was visually inspected by the female delivery; and the number of pups per litter was counted.

For analysis of blood testicular barrier (BTB) integrity, 15 μL of EZ-Link Sulfo-NHS-LC-Biotin (7.5 mg/mL; Thermo Fisher Scientific, Brebières, France) were injected into the left testis of anesthetized males exposed to a HFD or HF-CA diet [5]. Then, after 20 min., testes were removed, PFA-fixed and embedded in paraffin, and 5-μm-thick sections were prepared and stained for biotin.

### Histology

TUNEL and Ki67 experiments were performed as reported in [8], [9]. We performed the cell counts in two to three independent experiments with at least 5 mice per group. In addition, for each male, counting was made on 2 non following slides.

Testis weight, the number of tubules per section and the evaluation of tubule diameter were not affected between groups. As all these parameters are equal for the different groups there might be no bias in the count of positive cells or tubes.

### TUNEL analysis

Five-micrometer-thick paraffin-embedded sections were deparaffined with toluol followed by rehydratation. The slides of each group were incubated for 5 min in unmasking buffer (citrate acetate 1.8 mm, sodium citrate 8.2 mm, pH 6.0) at 86 C. Then the slides were incubated with 0.3 U/μl terminal deoxynucleotidyl transferase (Euromedex, Mundolsheim, France), 6.7 mm biotin-11-dUTP (Euromedex), and 26.7 mm dATP (Promega, Charbonnières, France) in terminal deoxynucleotidyl transferase buffer 1 h at 37 C. For the negative control, the enzyme was omitted. Extravidin alkaline phosphatase conjugate (dilution 1:100; Sigma-Aldrich) was added onto the slides for 25 min. Sigmafast FastRed TR/Naphthol AS-MX (Sigma-Aldrich) was used as the substrate according to the manufacturer’s instructions. Counterstain was performed with Mayer’s hematoxylin solution (Sigma-Aldrich) for 30 sec. In each testis, at least 100 random seminiferous tubules were counted. Results are expressed as the number of TUNEL-positive cells per 1000 seminiferous tubules. We perform the cell count (for TUNEL) in two to three independent experiments with at least 5 mice per group. In addition, for each male, counting was made on 2 non following slides.

### Ki67 counting

Five-micrometer-thick paraffin-embedded sections were fixed 10 min in 4% paraformaldehyde and washed three times for 10 min in 1× PBS. Cells were permeabilized with 0.1% Triton X–100 and 0.1% citrate solution in PBS for 2 min at 4 C. Slides were incubated with anti Ki67 1/500 (Tebu-bio, Le Perray en Yvelines, France) overnight at 4 C and then washed three times in 1× PBS. Slides were incubated for 1 h at room temperature with a goat anti-rabbit secondary antibody labeled with Alexa 488 (1/250; from Invitrogen Detection Technologies, Cergy-Pontoise, France). In each testis, at least 100 random seminiferous tubules were counted. Results are expressed as the number of Ki67-positive cells per 100 seminiferous tubules.

### Immunohistochemistry

Paraffin sections of PFA-fixed testis were sectioned at 5 μm. The sections were mounted on positively charged glass slides (Superfrost plus, Thermo Scientific, France), deparaffinized, rehydrated, treated for 20 min at 93–98°C in citric buffer (0.01 M, pH 6), rinsed in osmosed water (2 x 5 min) and washed (2 x 5 min) in Tris-buffered saline. Immunohistochemistry was conducted according to the manufacturer’s recommendations, as described earlier [10]. Slides were then counterstained with Hoestch medium (1 mg/ml). The antibodies used are given in supplemental information (S1 Table).

### Endocrine Investigations

Steroids were extracted from frozen testis (20 mg) with 10 vol of ethylacetate-isooctane (30:70, v:v) as previously described [9], and were measured using commercial kits: testosterone (Diagnostic Biochem, London, Canada).

Cholesterol, cholesterol esters and triglycerides were extracted from frozen testis as described previously with Folch method [11]. Cholesterol and cholesterol ester measurements were performed as recommended by manufacturer (Cholesterol and cholesterol ester: Calbiochem 428901; Triglycerides: Diagnostic Sys-tem, Holzheim Germany).

Glucose was measured on plasma upon manufacturer recommendations (Biomerieux 61269).

### Real-Time RT-PCR

RNA from testis samples were isolated using Nucleospin RNA (Macherey-nagel, Hoerdt, France). cDNA were synthesized from total RNA with the MMLV reverse transcriptase and random hexamer primers (Promega, Charbonnière Les Bains, France). The real-time PCR measurement of individual cDNAs was performed using SYBR green dye (Master mix Plus for SYBR Assay, Eurogentec, Angers, France) to measure duplex DNA formation with the Eppendorf Realplex system. The sequences of primers are reported in S2 Table. Standard curves were generated with pools of testis cDNA from animals with different treatments. The results were analyzed using the ΔΔct method.

### Western blot

Proteins were extracted from tissues using lysis buffer (0.4 M NaCl, 20 mM Hepes, 1.5 mMMgCl_2_, 0.2 mM EDTA, 0.1% NP40, 1X protease inhibitors (Roche Diagnostics, Meylan, France)). Antibodies were used in TBS, 0.1% tween and 10% milk. The antibodies used are given in S1 Table.

### Statistics

Differences between *two groups* for single point data were determined by Student’s *t*-test. All numerical data are represented as mean ± SE. Significant difference was set at *P* < 0.05.

## Results

### BA-exposure improves metabolic abnormalities induced by HFD

**Fig 1:**
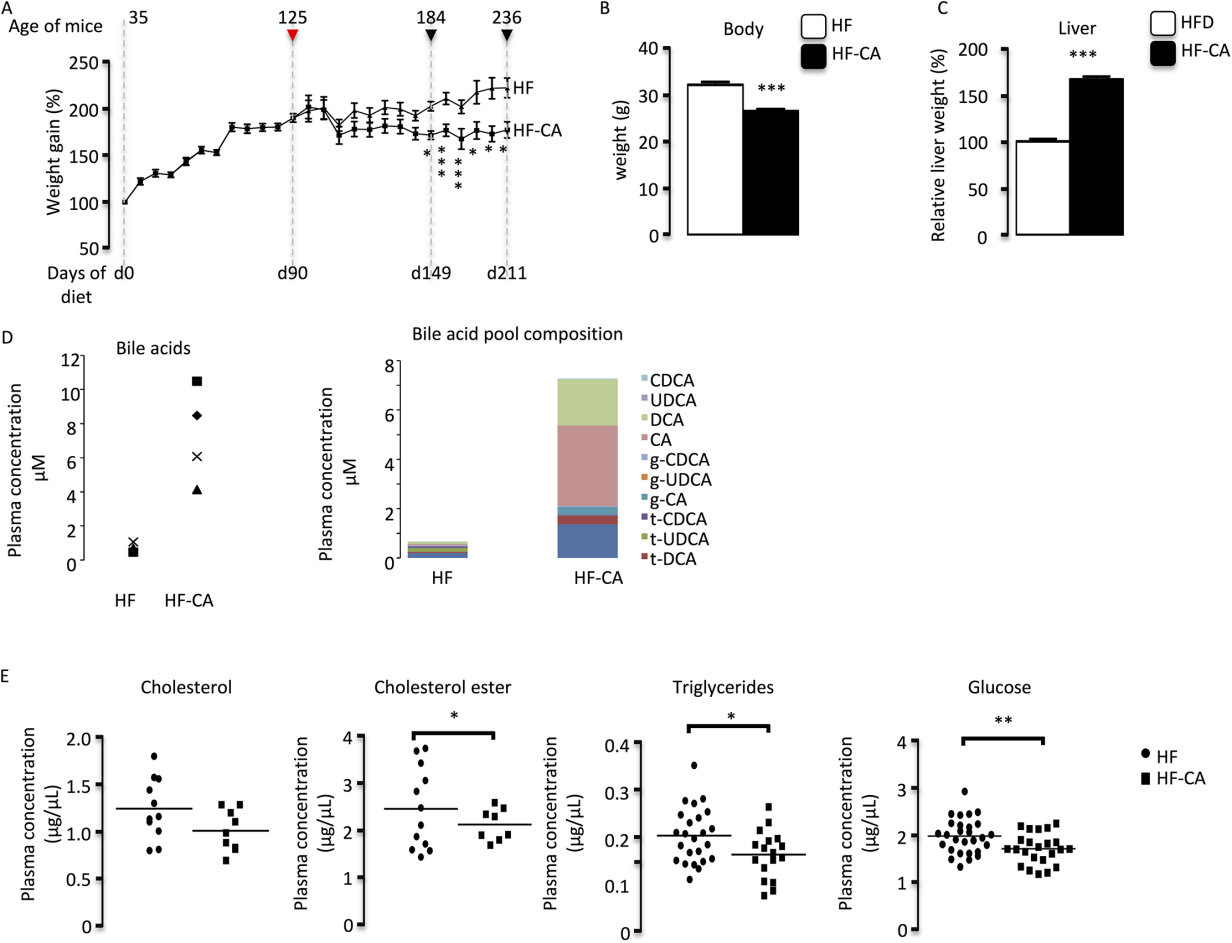
CA-supplementation reverse HFD induced overweight. **(A)** Weight gain of C57BL/6J mice along the experiments. After 90 days of high fat diet (HFD) (red arrow), half of the mice on the HFD (triangles) were switched to HFD supplemented with CA (HF-CA) (squares) (n = 12–35 per group). Black arrows indicated the timing of fertility test. **(B)** Relative body weight 4 months after the switch to HF-CA diet. **(C)** Relative liver weight normalized to body weight in C57Bl/6 mice fed HFD and HF-CA diet 4 months after the switch to HF-CA diet. (n = 18–25 per group). **(D)** Plasma bile acid levels and pool composition in mice under HFD or HF-CA diet 4 months after the switch to HF-CA diet. (**D)** Plasma cholesterol, cholesterol ester, triglycerides and glucose levels in mice fed to HFD or HF-CA diets 4 months after the switch to HF-CA diet. (n = 19–25 per group). Data represent mean ± SEM; Statistical analyses: * p<0,05; ** p<0,01 and *** p<0,001.

In order to understand the potential impact on male fertility of long term treatment of MetS diseases with molecules that activate bile acid signaling pathways, male mice were exposed to high fat diet (HFD) and then to HFD supplemented with 0.5% cholic acid (HF-CA) as previously described by Watanabe et al. [7]. Male mice exposed to HFD showed increased body weight superior to animal under chow diet throughout the experiment **(S1C Fig),** validating our model to induce mouse overweight. In addition, HFD induced increased levels of cholesterol, cholesterol ester, and glucose **(S1D Fig).** Once the overweight is established, part of the mice fed HFD was shifted to HF-CA to reverse the obesity. As expected, these mice showed a decreased of body weight gain compared to HFD group that is significant at 2 and 4 months after the switch to HF-CA diet (**S1D Fig** and **Fig 1A & 1B**). In accordance with bile acid effects [5], liver weight was increased in BA-exposed group compared to HFD group since 2 months after diet switch **(S1E Fig)** and still increased after 4 months (**Fig 1C**). Plasma BA levels were highly increased in HF-CA group compared to HFD group **(Fig 1D)**. The analysis of the BA pool revealed the increase concentrations of CA and DCA species **(Fig 1D)**. As expected [4], HF-CA diet led to a decrease of plasma total cholesterol and cholesterol esters and triglyceride levels **(Fig 1E)**. Accordingly, the impact of the HF-CA diet was confirmed at the glucose levels which were lower compared to HFD group (**Fig 1E**). Note that for some of these plasma parameters effects were observed since 2 months after the switch to HF-CA diet **(S2A Fig).**

### BA-exposure alters male fertility in the context of metabolic syndrome

**Fig 2:**
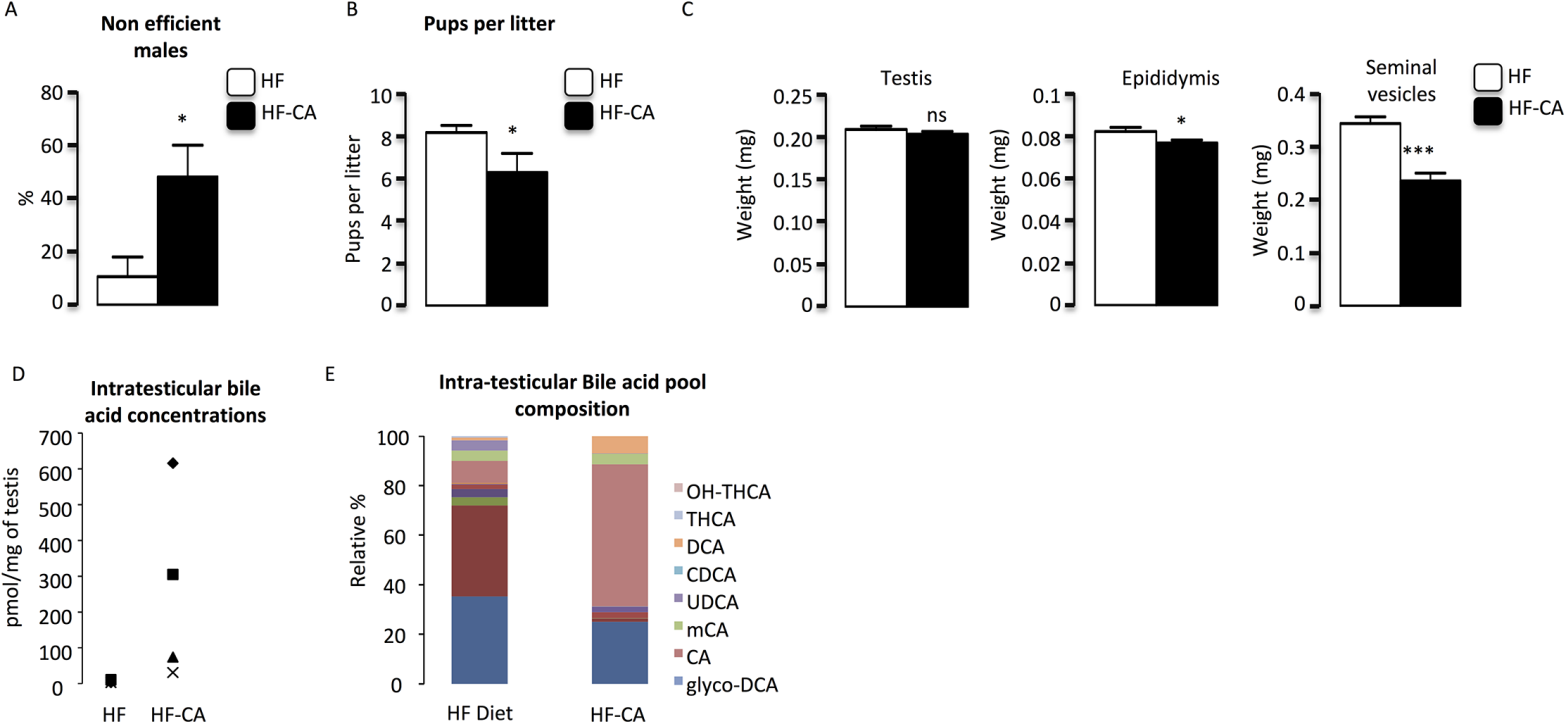
CA-supplementation alters male reproduction function in mice fed HF-CA diets versus HF-diet. **(A)** Percent of non-efficient males after 15 days of breeding with 2 C57BL/6J females in mice under HFD or HF-CA diet 4 months after the switch to HF-CA diet. **(B)** Number of pups per litter obtained in mice under HFD or HF-CA diet 4 months after the switch to HF-CA diet. **(C)** Relative testis, epididymis and seminal weights normalized to body weight in C57Bl/6 mice fed HF-diet and HF-CA diet 4 months after the switch to HF-CA diet. (n = 18–25 per group). (**D)** Intra-testicular bile acid levels and pool composition in mice under HFD or HF-CA diet 4 months after the switch to HF-CA diet. Data represent mean ± SEM; Statistical analyses: * p<0,05; ** p<0,01 and *** p<0,001.

As BA exposure was previously demonstrated to induce male fertility disorders, we next analyzed male reproductive capacity. Male fertility was assessed by breeding males of each group with C57Bl6J females during 2 weeks (as described in method section). Fertility tests highlight reproductive defaults in HF-CA group compared to HFD group (**Fig 2A**). Even if there was no difference in the number of vaginal plugs observed per male between groups (data not shown), data revealed a 4 to 5-fold increase in the number of males unable to give progeny after two weeks of breeding with females; these males were thus qualified as non-efficient males (**Fig 2A**). For the males that were still able to give progenies, there was a significant decrease in the number of pups per litter in HF-CA animals (**Fig 2B**). No significant effect of CA-supplementation was observed on fertility parameters 2 months after the switch to HF-CA diet **(S2B Fig).**

This impact on fertility was sustained by lower weight of organs of genital tract such as epididymis and seminals in HF-CA treated males compared to HFD group (**Fig 2C and S2C Fig**). Note that no effect was observed on testis weight (**Fig 2C**).

We then analyzed whether bile acid levels and pool were modified in HF-CA group compared to HFD males. We show a 6-fold increase of BA concentrations in testis of males exposed to HF-CA diet compared to HFD group **(Fig 2D)**. This is consistent with what was previously published by Baptissart et al. [5]. Consistently, BA pool analyses revealed the increase concentration of CA and DCA species **(Fig 2E)**.

### BAs induce lower germ cell proliferation rate

**Fig 3:**
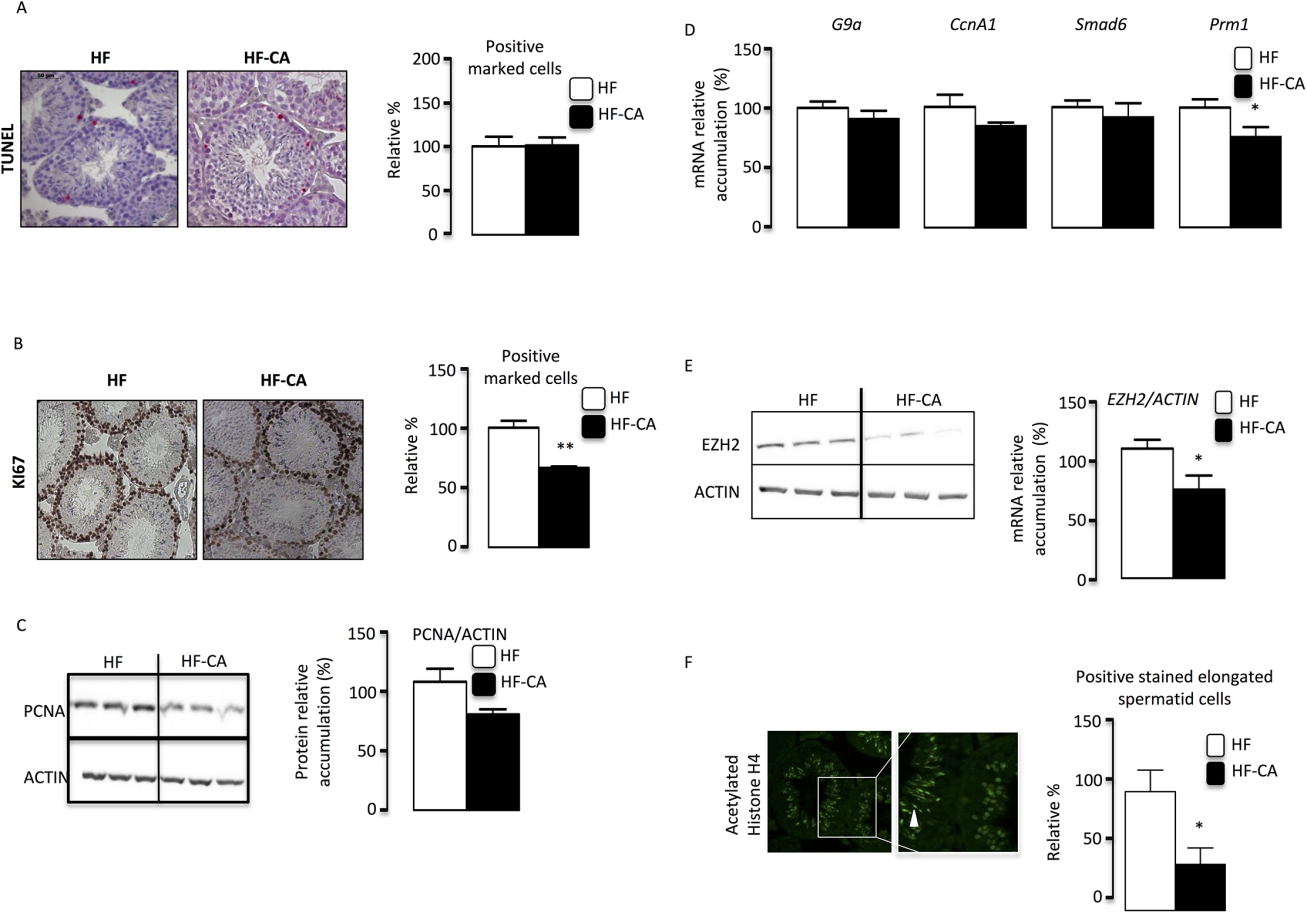
CA-supplementation diet induces increase of germ cell proliferation. **(A)** Apoptosis in mice exposed to HFD or HF-CA diets 4 months after the switch to HF-CA diet (n = 13 to 25 per group) analyzed by TUNEL staining. The arrow indicates apoptotic spermatocytes. The original magnification was X200. The number of TUNEL-positive cells per 100 seminiferous tubes. **(B)** Proliferation in mice exposed to HFD or HF-CA diets (n = per groups) analyzed by Ki–67 staining. Representative micrographs of the testis exposed to HF and HF-CA diets. The original magnification was X100.Quantification of the number of Ki-67-positive cells per 100 seminiferous tubules after 2 and 4 months of HF and HF-CA diets (n = 4–5 per groups). **(C)** Immunoblot of PCNA and ACTIN on testicular protein extracts of HFD or HF-CA diet 4 months after the switch to HF-CA diet (n = 5–8 per groups). Quantification of PCNA/ACTIN ratio. HFD group was arbitrarily fixed at 100%. **(D)** Testicular mRNA expression of *G9a*, *Cyclina1 (Ccna1)*, *Smad6* and *Prm1* normalize to β-actin levels in whole testis of C57BL/6 mice fed HFD or HF-CA diets 4 months after the switch to HF-CA diet (n = 16–22 per groups).). **(E)** Immunoblot of EZH2 and ACTIN on testicular protein extracts of HF diets or HF-CA diets 4 months after the switch to HF-CA diet (n = 5–8 per groups). Quantification of EZH2/ACTIN ratio. HFD group was arbitrarily fixed at 100%. **(F)** Evaluation of post-meiotic elongated spermatids in mice exposed to HFD or HF-CA diets analyzed by acetylated histone H4 (H4ac) staining 4 months after the switch to HF-CA (Representative micrographs of the testis exposed to HF diet). The original magnification was X100.Quantification of the number of H4ac-positive cells (n = 4–5 per groups). Data represent mean ± SEM; Statistical analyses: * p<0,05; ** p<0,01 and *** p<0,005.

HF-CA diet did not drastically alter histology of testis compared to HFD group (data not shown). No statistical impact of HF-CA-diet was observed on germ cell apoptosis compared to HFD-group (**Fig 3A**). However, we confirmed that as previously described HFD induced germ cell apoptosis (**S3A Fig**). In contrast, a significant decrease of germ cell proliferation was observed at 4 months in HF-CA group compared to HFD-group as identified by Ki67 staining (**Fig 3B**). This decrease in germ cell proliferation was supported by the lower accumulation of the PCNA protein in HF-CA group compared to HFD animals (**Fig 3C**). This data suggests that there could be some abnormalities in spermatogenesis process. In order to define which spermatogenesis step might be altered, we performed analysis of mRNA accumulation of specific markers for early spermatogenesis (G9a), spermatocytes (Cyclin-a1), round spermatids (Smad–6) and late germ cells (Protamin–1; Prm–1). Results show a lower expression of *Prm–1* a gene referred to be expressed in elongating/elongated spermatids **(Fig 3D)**. This is also sustained by the lower EZH2 protein level, a marker of post-meiotic germ cells [12], in HF-CA group compared to HFD animals **(Fig 3E)**. The decrease of number of post-meiotic cells within the testis exposed to HF-CA diet was then validated by immunohistochesmistry with acetylated-histone H4 staining and the identification of a lower the number of positive cells per tubes in HF-CA group compared to HFD exposed males **(Fig 3F)**. No difference was observed regarding intra-testicular levels of some lipid parameters such as cholesterol, cholesterol ester or triglycerides either 2 or 4 months after the switch to HF-CA diet **(S3B and S3C Fig)**.

### BA exposure leads to altered testicular endocrine function

**Fig 4:**
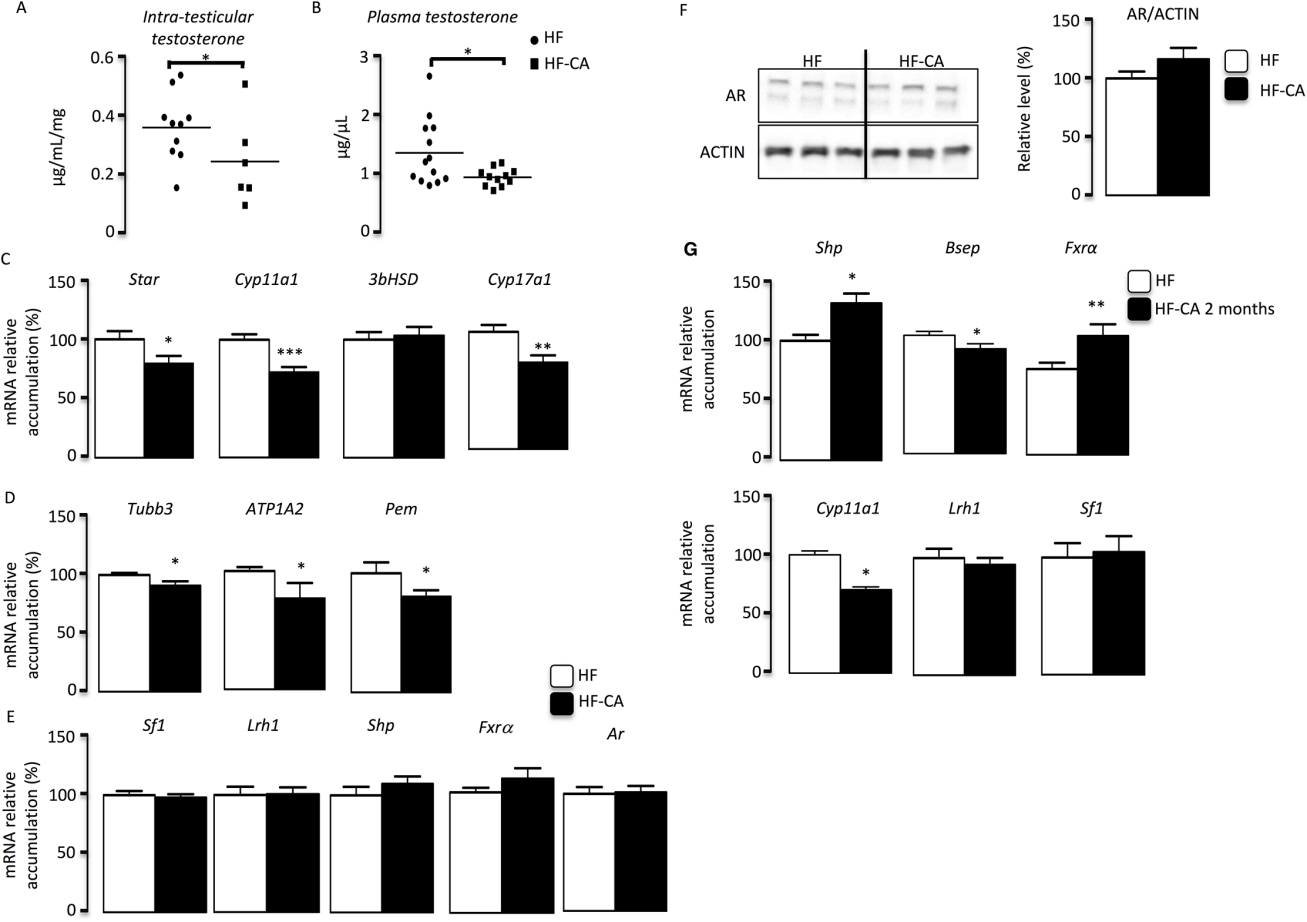
CA-supplementation alters testicular endocrine function. **(A)** Testicular testosterone levels in mice fed HFD and HF-CA diet 4 months after the switch to HF-CA diet. (n = 7–13 per group). (**B)** Plasma testosterone levels in mice fed HFD and HF-CA diet 4 months after the switch to HF-CA diet. (n = 7–13 per group).**(C)** Testicular mRNA accumulation of *Star*, *Cyp11a1*, *3βHDS* and *Cyp17a1* normalized to *β-actin* mRNA levels in the whole testes of mice fed HFD and HF-CA diet 4 months after the switch to HF-CA diet (n = 12–22 per group). **(D)** Testicular mRNA expression of *Tubb3*, *Atp1a2* and *Pem* normalized to *βactin* m in the whole testes of mice fed HFD and HF-CA diet 4 months after the switch to HF-CA diet (n = 7–22 per group). **(E)** Testicular mRNA expression of *Sf–1*, *Lrh–1*, *Shp*, *Fxrα* and *Ar* normalized to *β-actin* m in the whole testes of mice fed HFD and HF-CA diet 4 months after the switch to HF-CA diet (n = 7–22 per group). **(F)** Immunoblot of AR and ACTIN on testicular protein extracts of HFD or HF-CA diet 4 months after the switch to HF-CA diet (n = 5–8 per groups). Quantification of AR/ACTIN ratio. HF-diet group was arbitrarily fixed at 100%.HF-diet group was arbitrarily fixed at 100%. **(G)**. Testicular mRNA expression of *Shp*, *Bsep*, *Fxrα*, *Cyp11a1*, *Lrh–1*, Sf–1 and normalize to β-actin levels in whole testis of C57BL/6 mice fed HFD or HF-CA diet 2 months after the switch to HF-CA diet (n = 16–22 per groups. Data represent mean ± SEM; Statistical analyses: * p<0,05; ** p<0,01 and *** p<0,005.

Testicular physiology has been demonstrated to be highly dependent of steroid metabolism as highlighted by the numerous data coming from studies on endocrine disrupters. 4 months after the switch to HF-CA diet; CA supplementation alters testicular steroidogenesis as highlighted by the lower intra-testicular and plasma testosterone levels in HF-CA diet group compared to HFD group (**Fig 4A & 4B**). Interestingly, FXRα has been previously demonstrated to repress testicular steroidogenesis via the induction of SHP which in turn inhibits *Sf–1* and *Lrh–1* expression and their transcriptional activities [6], [13]. The potential activation of the FXRα signaling pathways within the testis in the HF-CA context was assessed through the analysis of FXRα and two of its target genes *Shp* and *Bsep*. Shp was not affected after 4 months of diet (**Fig 4E**). However, we found an increase of FXRα and Shp mRNA accumulation if HF-CA group compare to HFD group after two months of HF-CA diet exposure **(Fig 4G)**. In contrast, a lower mRNA accumulation of *Bsep* was observed after two months of HF-CA diet exposure **(Fig 4G).** This downregulation of *Bsep* must be correlated to the higher level of Shp as it was previously demonstrated that SHP inhibit the expression of *Bsep* through LRH–1 dependent activity [6]. The analysis of *Sf–1* and *Lrh–1* revealed that their expression levels were not affected in HF-CA exposed group compared to HFD animals at 4 months or 2 months after the beginning of HF-CA diet (**Fig 4E & 4G**). These data suggest that SHP might act mainly through the inhibition of the transcriptional activity of LRH–1 and SF–1 as previously suggested in some recent reports [8]. Even though short term experiments are need to clearly characterize the induction of FXRα pathways in the HF-CA context, we decided to explore the impact of HF-CA on testicular steroidogenesis. Indeed, regarding the testicular physiology, the only define pathway targeted by FXRα so far is the steroidogenesis. This effect was published to be dependent of SHP [6]. In that line, we define that after two months of HF-CA diet the expression of steroidogenic gene *Cyp11a1* was decreased (Fig 4G). In addition the decreased testicular testosterone levels after 4 months of HF-CA diet was correlated with the impact on the mRNA accumulation of genes involved in steroid synthesis such as *Star*, *Cyp11a1* and *Cyp17a1* (**Fig 4C**). Furthermore, these results were associated with decreased expression levels of several androgen-dependent genes (*Tubb3*, *Atp1a2*, *Pem*) four months after the beginning of HF-CA diet (**Fig 4D**). This impact on androgen-dependent genes was not due to alteration of the expression of the androgen receptor neither at the mRNA level (**Fig 4E**) nor at the protein level (**Fig 4E**).

### BAs alter seminiferous epithelium integrity

**Fig 5:**
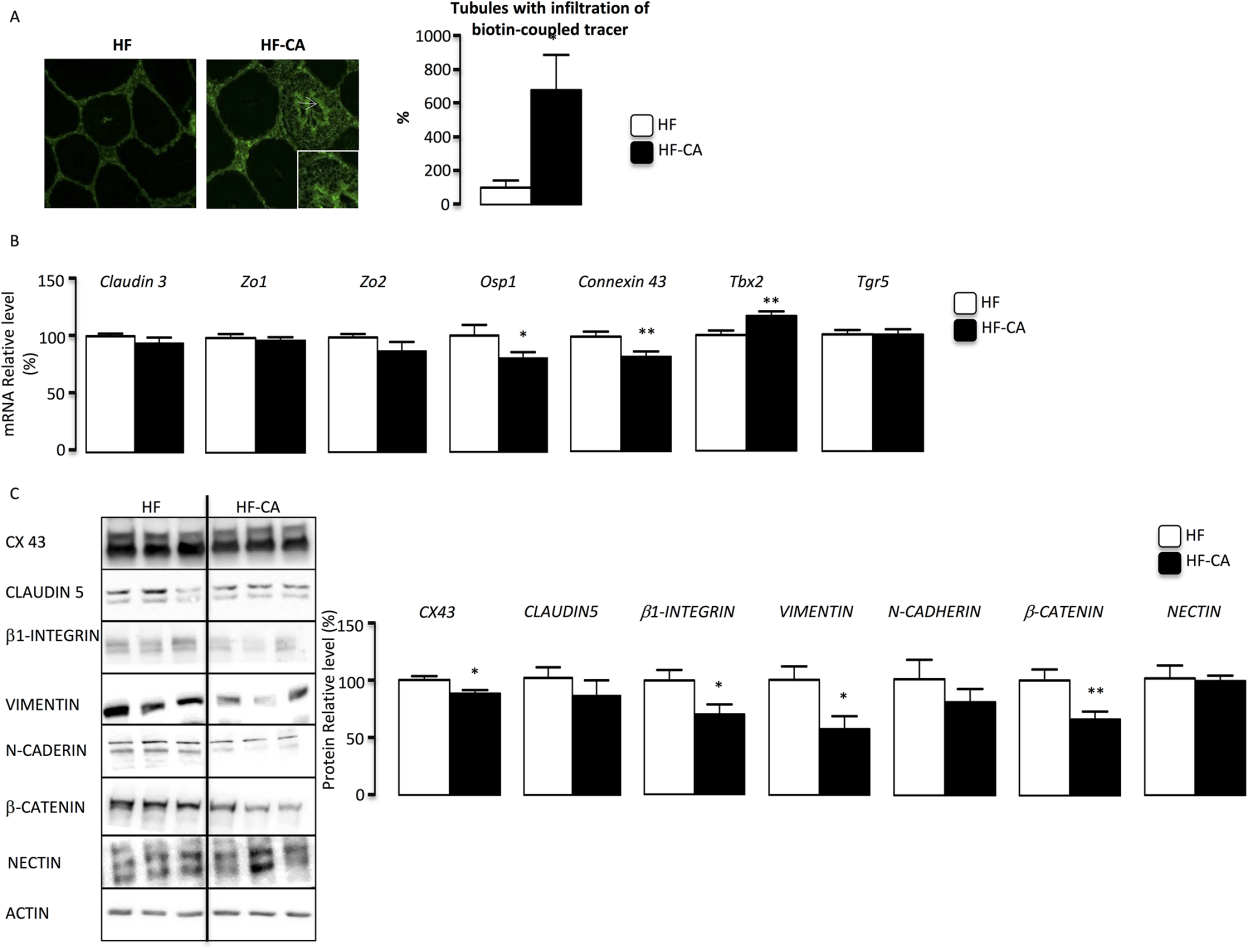
CA-supplementation alters seminiferous epithelium. **(A)** Blood-testis barrier integrity as measured by the stained testis for EZ-link-biotinylated. Representative micrographs of mice fed HFD or HF-CA diet 4 months after the switch to HF-CA diet. The arrow indicates a tubule with a high intensity of infiltration. The original magnification was X100. Quantification of the number of tubules with infiltration per 100 seminiferous tubules (n = 5–12 per group). **(B)** Testicular mRNA expression of *Claudin–3*, *Zo1*, *Zo2*, Osp1,*Cx 43*, *Tbx2 and Tgr5* normalize to β-actin levels in whole testis of C57BL/6 mice fed HFD or HF-CA diet 4 months after the switch to HF-CA diet (n = 16–22 per groups). **(C)** Immunoblot of CONNEXIN43, CLAUDIN5, INTEGRINβ1, VIMENTIN, N-CADHERINE, β-CATENINE and NECTIN on testicular protein extracts of HFD or HF-CA diet for 4 months (n = 5–8 per groups). Quantifications of proteins of interest were made normalized to ACTIN. HF-diet group was arbitrarily fixed at 100%. Data represent mean ± SEM; Statistical analyses: * p<0,05; ** p<0,01 and *** p<0,005.

We have recently identified that BA impact testicular physiology through TGR5 dependent mechanisms [5]. TGR5 activation leads to the control of the TBX2-Cx43 pathways which is associated with the alteration of the blood-testis-barrier (BTB). The BTB is a critical structure of the seminiferous epithelium and has been demonstrated to be altered following the increase of BA levels. Here, we show that the BTB is no longer intact after 4 months of HF-CA diet, as shown by the use of biotin-coupled tracer (**Fig 5A**). Consistent with what was previously published, bile acid exposure decreased accumulation of *Connexin–43* at both mRNA and protein (**Fig 5B & 5C**). This was associated with the increase of *Tbx2* mRNA accumulation in HF-CA group compared to HFD after 4 months of diet exposure (**Fig 5B**). These data suggest that TGR5 pathways might have been induced by CA-supplementation. However, no effect of HF-CA diet was observed on TGR5 mRNA expression (**Fig 5B**). Regarding the expression of other genes involved in cell-cell interactions, a lower mRNA accumulation of *Osp1* in BA-fed mice was observed (**Fig 5B**). In contrast, no effects were observed regarding the mRNA accumulation of *Zo1*, *Zo2* and *Claudin3* (**Fig 5C**). No effect was observed on CLAUDIN–5, NECTIN protein accumulations (**Fig 5C**); however we confirmed the impact of cell-cell interactions within the seminiferous tubule as the protein accumulations of INTEGRIN-β1, VIMENTINE, N-CADHERIN and β-CATENINE was decreased in HF-CA exposed males compare to HFD group (**Fig 5C**).

## Discussion

Recently, we showed that adult mice fed a diet supplemented with CA have altered fertility subsequent to testicular defects [5]. The use of BA supplementation has been demonstrated to be able to reverse obesity induced by HFD in mice. In the following years, subsequent studies highlighted the links between BA exposure and improvement of diseases such as diabetes.

We thus wonder what could be the consequences on male fertility of a long term exposure to BA derivatives in the treatment of MetS. To investigate these potential links, we have reproduced the protocol of Watanabe and collaborators [7]. Male mice were exposed to HFD to induce overweight and use supplementation with 0.5% cholic acid to reverse the obesity.

The present work confirms the deleterious impact of BA exposure on male fertility even during the treatment of MetS. Indeed, the BA-supplemented diet led to an increase of BA concentrations in plasma and testis. The composition of BA species within the testis in HF-CA exposed males are comparable to what was previously published by Baptissartwith the increase of CA and DCA species.

The complexity of the interactions between metabolism and male fertility has been described among the last decades [14]. Among all the consequences of MetS, male reproductive function is altered in some obese man patients. This is highlighted by the increasing evidence of an interactions between MetS and testicular functions [14]. However, the relation between MetS and male reproductive function remains to be fully deciphered.

This complexity still need to be highly studied as we demonstrate here that even with the amelioration of several global health parameters such as plasma cholesterol, triglycerides and glucose male fertility remain altered. In that line BA-diet have no impact on metabolic parameters (glucose, cholesterol, triglycerides) in the testis, it reverse the impact of HF diet on these parameters at the plasma level. Thus our data reinforce the links between BA signaling pathways and testicular physiology and pathophysiology. However, it could not be excluded that their might be direct impact of BA-exposure on organs involved in post-testicular maturation of the spermatozoa such as the epididymis and the seminals. Such effects should be studied in future works as the decrease of seminal weights under BA-diet conditions are reported here and in previous report [5].

Even though the clear involvement of systemic or local testicular action of BA receptors cannot be established there as specific testicular invalidation would have been required, we demonstrate that BA exposure impact testicular physiology in HF-CA context. Our goal here was to define if alterations induced by BA-exposure, as previously published, are reproducible in HF context.

Previous report highlights the major role of TGR5 regarding the impact of BA on male fertility during cholestasis. The results of the present study supported the activation of the TGR5 signaling pathways in testis following HF-CA diet exposure as we do observed as previously published [5] the increase of *Tbx2* mRNA accumulation and the decrease of CX43 at both mRNA and protein levels.

However, the present work clearly suggests that in the context of MetS, the involved mechanisms are more complex than previously describe. Indeed, in contrast to the study from Baptissart et al., HF-CA diet leads to a deregulation of testicular steroidogenesis. Such effect could be consistent to some of the effect of the FXRα signaling pathway within the testis as the use of GW4064, a FXRαsynthetic agonist, was demonstrated to repress testosterone synthesis in a SHP dependent manner [6]. In that line we do observed that after HF-CA administration the expression of SHP was increased with a decrease of steroidogenic genes and then testosterone levels. In addition the lower mRNA accumulation of *Bsep* was observed after two months of HF-CA diet exposure **(Fig 4G).** This downregulation of *Bsep* must be correlated to the higher level of Shp as it was previously demonstrated that SHP inhibit the expression of *Bsep* through LRH–1 dependent activity [6].

Here, no difference in the status of germ cell apoptosis was observed between HFD and HF-CA groups; this is in contrast with previous data showing apoptosis of post-meiotic germ cells (spermatids) during liver disease induced by BA exposure [5]. This might be due to the fact that HFD *per se* induces apoptosis of spermatocytes cells (**S3A Fig**), which are earlier germ cell steps than spermatids, this effect of HFD might mask the impact of BA on spermatids. However, a lower production of post-meiotic germ cells in HF-CA group is sustained by our results showing lower accumulation of post-meiotic germ cells markers at either mRNA and protein levels.

**Fig 6:**
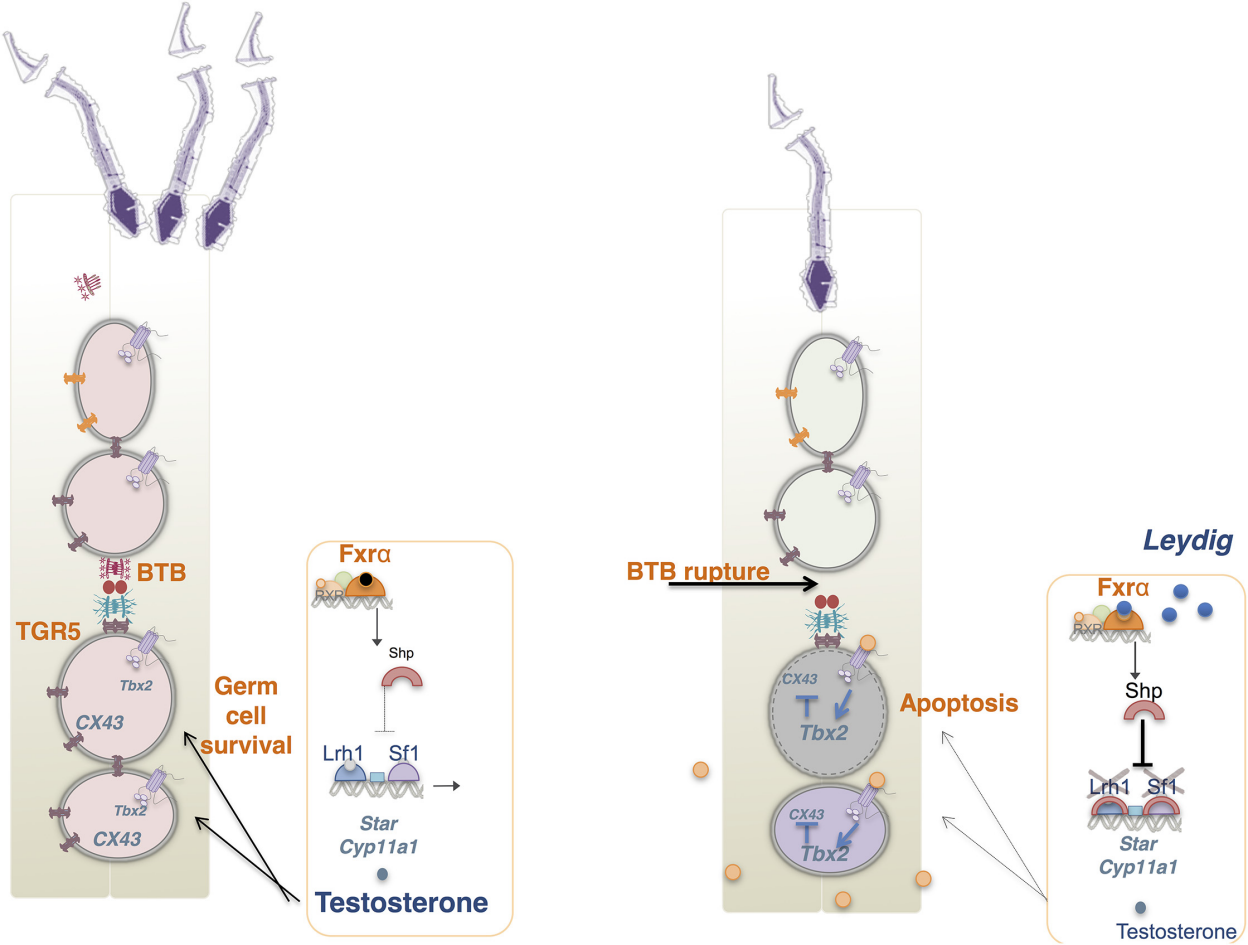
Potential intratesticular action of BAs. In normal condition production of testosterone is involved in germ cell survival. The expression of genes involved in steroidogenic pathway is in part supported by transcriptional activity of LRH–1 and SF1. In parallel, the integrity of the seminiferous epithelium is ensured by the establishment of cell-cell interactions involving protein such as Cx43. In the context of BA exposure, lower production of testosterone is observed. This is consistent with the potential activation of the FXRαwhich in turn leads to activation of SHP a known repressor of steroidogenic pathways via the inhibition of transcriptional activity of LRH–1 and SF–1 on promoter sequences of genes such as Star or Cyp11a1. In parallel, in BA context, the integrity of blood testis barrier is altered. This is consistent with the activation of the TGR5-Tbx2 signaling pathways leading to lower accumulation of protein involved in cell-cell interactions that destabilized the structure of the seminiferous epithelium. These alterations might participate to the increase rate of germ cell apoptosis within the testis. In regards to the major role of testicular physiology, even though post-testicular impact cannot be exclude; the present work suggests that this alterations of testicular physiology induced in a HF-diet context might participate to the appearance of male infertility.

The present work suggests that in the pathophysiological model of HF-diet, several BA signaling pathways among which either FXRα or TGR5 could be activated. Additional studies must be performed using specific agonists of FXRα and TGR5 to better decipher the critical pathways involved. Using an integrative scheme, we try here to summarize the potential involvement of both signaling pathways within the testis **(Fig 6)**.

In addition, the present results on the impact of BA exposure in context of HFD on fertility could be useful to understand some unexplained rare clinical situations. This could be consistent with a case report data showing subsequent infertility in some obese men treated with bariatric chirurgical approach [15]. It is of interest to note that in such treatment, BA levels have been demonstrated to be increased [16]. Even if it is with low incidence, our work could open new perspectives to better understand such rare pathological cases.

This work highlights the urgent need to clearly define the molecular mechanisms driving the deleterious impact of BAs on testis. Further studies will be necessary to better define the pharmacologic or genetic modulations of different bile acid receptors during the treatment of metabolic disorders in order to minimize the impact on male reproductive functions.

In conclusion, our study raises the question of the long term consequences of treatment with BA derivatives. Thus it will be necessary to plan future experiments to define the potential secondary impacts of the activation of BA signaling pathway as the use of derivates from BAs as therapeutic molecules are proposed today (metabolic syndrome, cancer).

## Supporting Information

S1 ARRIVE Checklist NC3Rs ARRIVE Guidelines Checklist. The ARRIVE guideline checklist for animal research: reporting in vivo experiments.
(DOCX)

S1 Fig (A). Quantification of the number of seminiferous tubules per testis slide in HFD and HF-CA fed animals 4 months after the switch to HF-CA diet. (B). Quantification of the diameter of seminiferous tubules per testis slide in HFD and HF-CA fed animals 4 months after the switch to HF-CA diet. (C) Weight gain of C57BL/6J mice along the experiments fed chow or HFD for 236 days. (D) Relative body weight 2 months after the switch to HF-CA diet. (E) Relative liver weight normalized to body weight in C57Bl/6 mice fed HFD and HF-CA diet 2 months after the switch to HF-CA diet. (n = 18–25 per group). (E) Plasma cholesterol, cholesterol ester and glucose levels in mice fed to chow or HFD (n = 19–25 per group). Data represent mean ± SEM; Statistical analyses: * p<0,05; ** p<0,01 and *** p<0,001. (PDF)

S2 Fig (A). Plasma cholesterol, cholesterol ester, triglycerides and glucose levels in mice fed to HFD or HF-CA diets 2 months after the switch to HF-CA diet. (n = 19–25 per group). (B) Percent of non-efficient males after 15 days of breeding with 2 C57BL/6J females to analyse their capacity to mate. (C) Number of pups per litter obtained. (D) Relative testis, epididymis and seminal weights normalized to body weight in C57Bl/6 mice fed HFD and HF-CA diet 2 months after the switch to HF-CA diet. (n = 18–25 per group). Data represent mean ± SEM; Statistical analyses: * p<0,05; ** p<0,01 and *** p<0,001. (PDF)

S3 Fig (A) Apoptosis in mice fed chow or HFD (n = 13 to 25 per group) analyzed by TUNEL staining. The arrow indicates apoptotic spermatocytes. The original magnification was X200. The number of TUNEL-positive cells per 100 seminiferous tubes. (B) Intra-testicular cholesterol, cholesterol ester, triglycerides levels in mice fed to HFD or HF-CA diet 2 months after the switch to HF-CA diet. (n = 19–25 per group). (C) Intra-testicular cholesterol, cholesterol ester, triglycerides and glucose levels in mice fed to HFD or HF-CA diet 4 months after the switch to HF-CA diet. (n = 19–25 per group). (PDF)

S1 Table The list of the antibodies used in the present work. (XLSX)

S2 Table The list of the primer sequences used in the present work. (XLSX)
