# Supplementary material for: Bile Acid Alters Male Mouse Fertility in Metabolic Syndrome Context
Source: PLoS One. 2015 Oct 6;10(10):e0139946. doi: 10.1371/journal.pone.0139946 (PMC4595338; doi:10.1371/journal.pone.0139946)

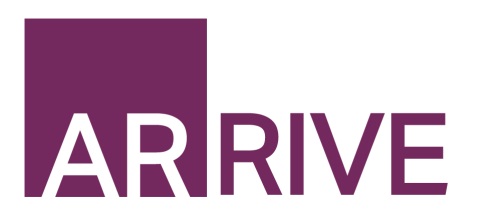


The ARRIVE Guidelines Checklist

Animal Research: Reporting In Vivo Experiments

Carol Kilkenny^1^, William J Browne^2^, Innes C Cuthill^3^, Michael Emerson^4^ and Douglas G Altman^5^

*^1^The National Centre for the Replacement, Refinement and Reduction of Animals in Research, London, UK, ^2^School of Veterinary Science, University of Bristol, Bristol, UK, ^3^School of Biological Sciences, University of Bristol, Bristol, UK, ^4^National Heart and Lung Institute, Imperial College London, UK, ^5^Centre for Statistics in Medicine, University of Oxford, Oxford, UK.*

|  | | ITEM | RECOMMENDATION | Section/ Paragraph |
| --- | --- | --- | --- | --- |
| 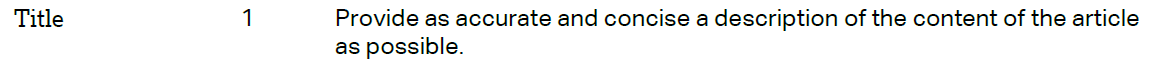 | | | Title |  |
| 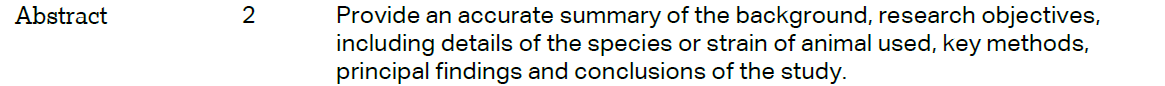 | | | Abstract |  |
| INTRODUCTION | | |  |  |
| 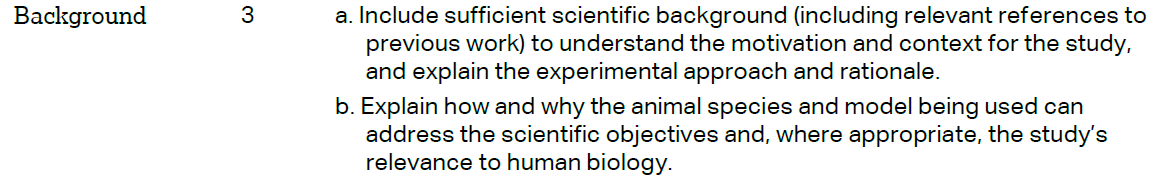 | | | Paragraph 2 to 5 |  |
| 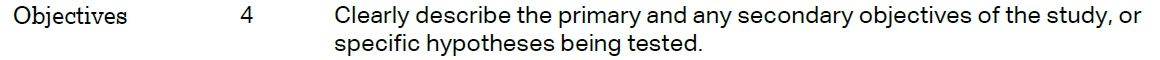 | | | Abstract  Paragraph 2 to 5 |  |
| METHODS | | |  |  |
| 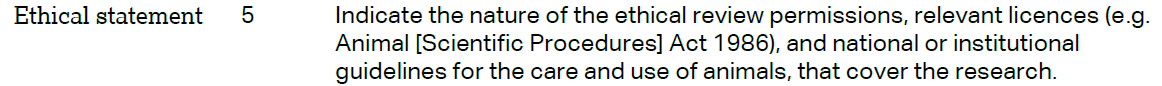 | | | Material and methods  Page 4 |  |
| 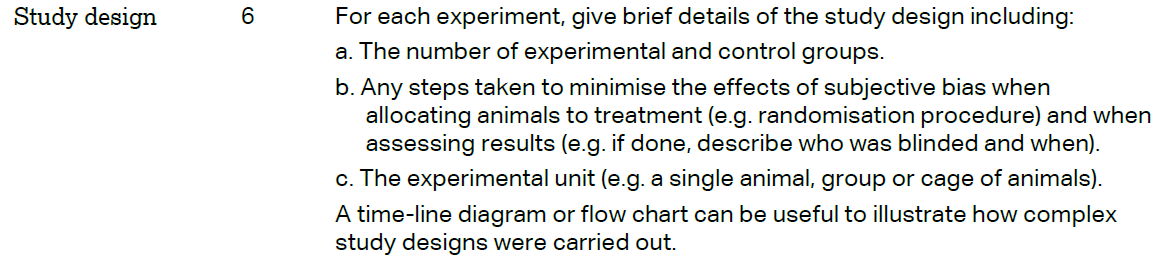 | | | Material and methods  Page 4-6 |  |
| 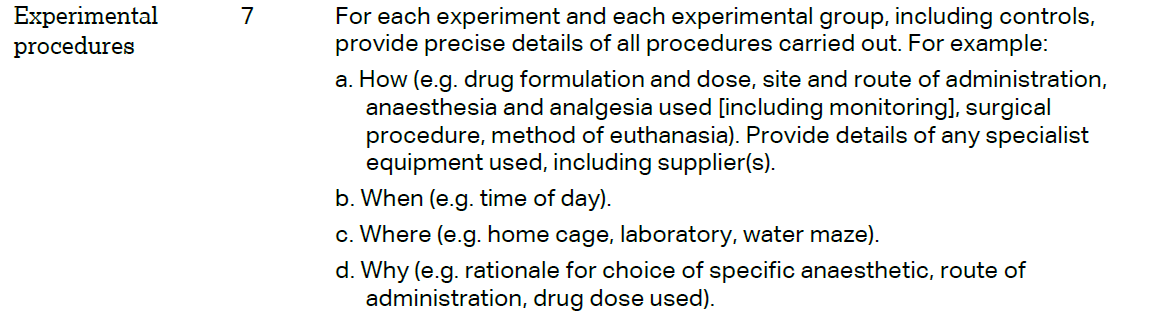 | | | Material and methods  Page 4 |  |
| 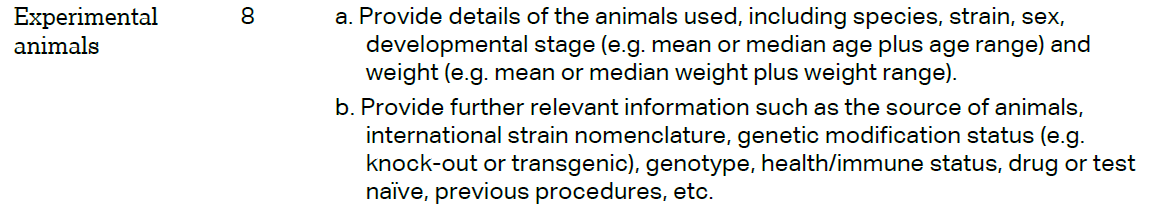 | | | Material and methods  Page 4 |  |

The ARRIVE guidelines. Originally published in *PLoS Biology*, June 2010^1^

| 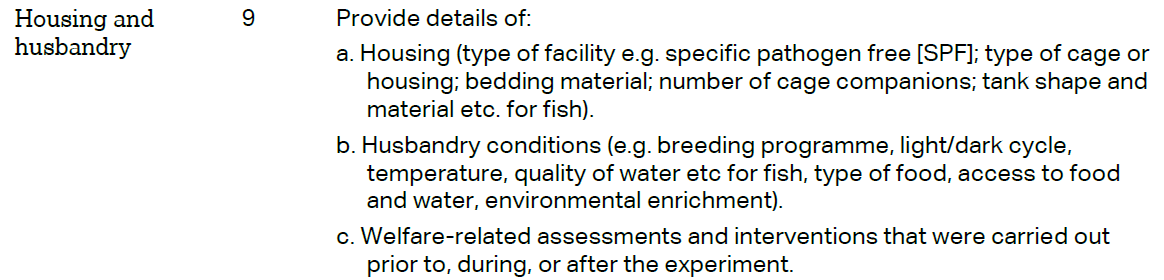 | Material and methods  Page 4 | |
| --- | --- | --- |
| 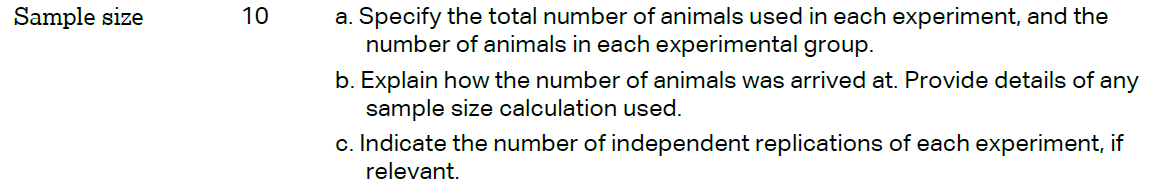 | Material and methods  Pages 4-6  Figure legends | |
| 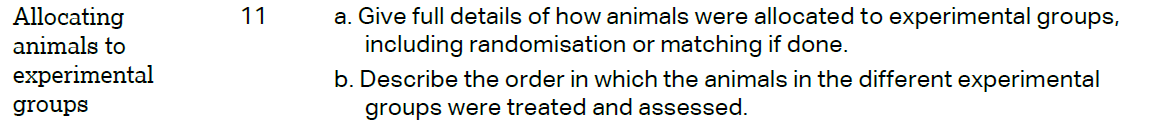 | Material and methods  Pages 4-6 | |
| 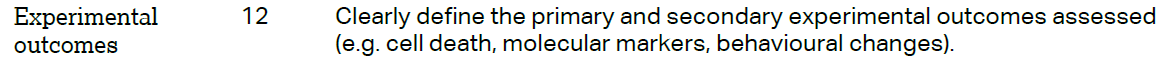 | Material and methods  Pages 4 to 6  Abstract | |
| 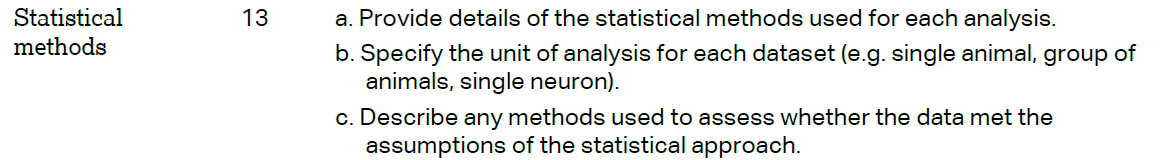 | Material and methods  Page 5 | |
| RESULTS |  | |
| 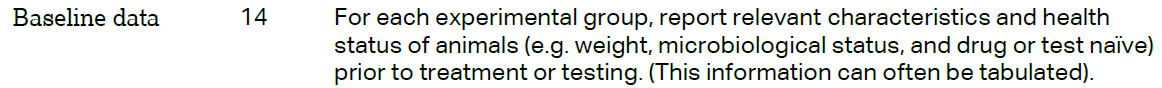 | Figure1 and texte results pages 6-7 | |
| 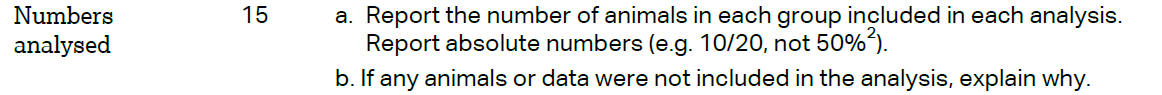 | Figure legends | |
| 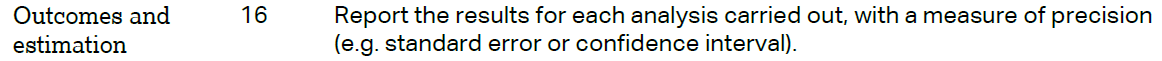 | Figure legends | |
| 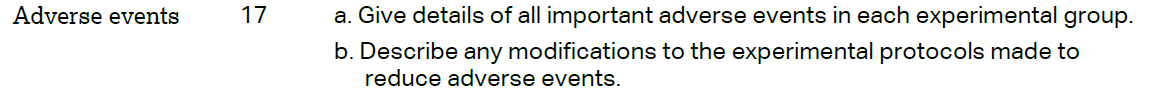 | None | |
| DISCUSSION |  | |
| 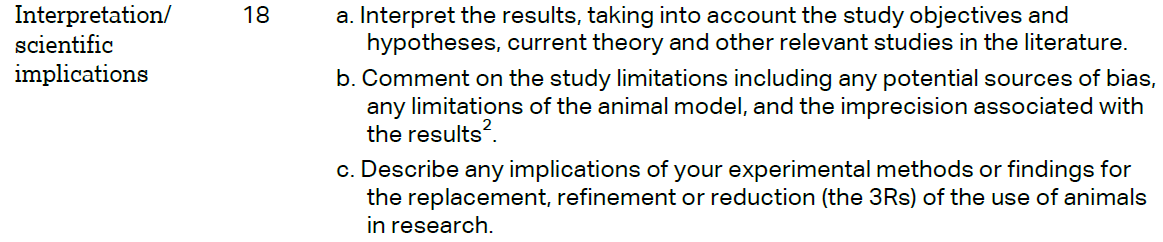 | Throughout results and discussion sections | |
| 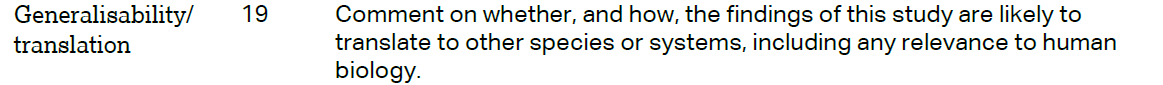 | Throughout discussion section | |
| 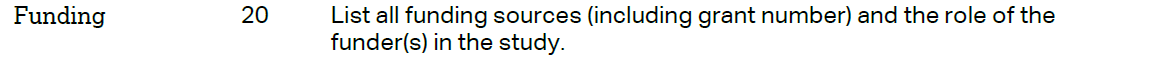 | | Page 1 |


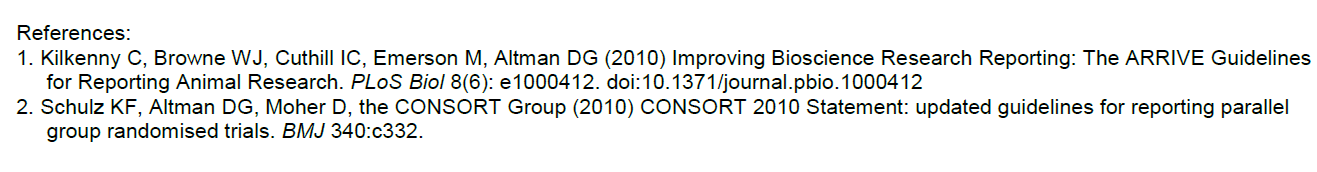

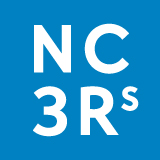

Supplement: S1 ARRIVE Checklist — The ARRIVE guideline checklist for animal research: reporting in vivo experiments. (DOCX) [file pone.0139946.s001.docx]
