# Supplementary figures and images for: Bile Acid Alters Male Mouse Fertility in Metabolic Syndrome Context
Source: PLoS One. 2015 Oct 6;10(10):e0139946. doi: 10.1371/journal.pone.0139946 (PMC4595338; doi:10.1371/journal.pone.0139946)

**A** Number of tubules per slide

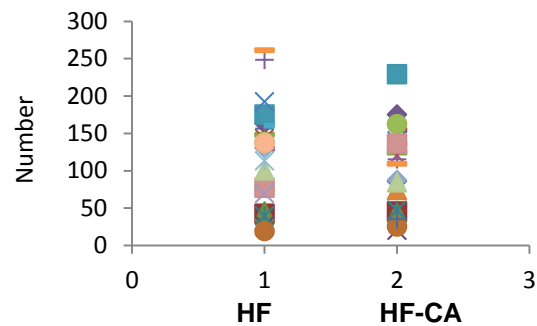

**B** Diameter of tubules

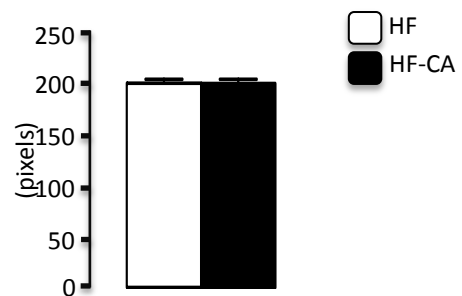

**C**

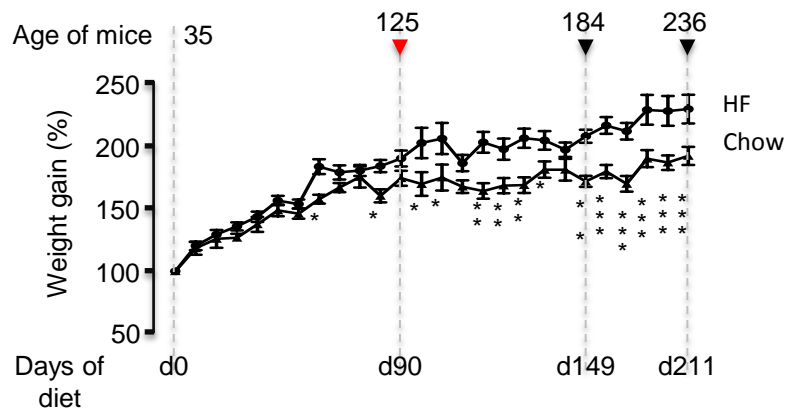

**D**

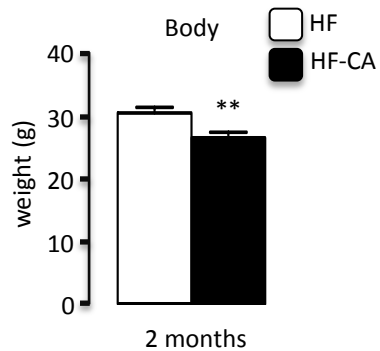

**E**

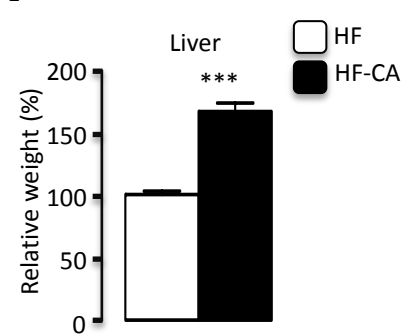

**F**

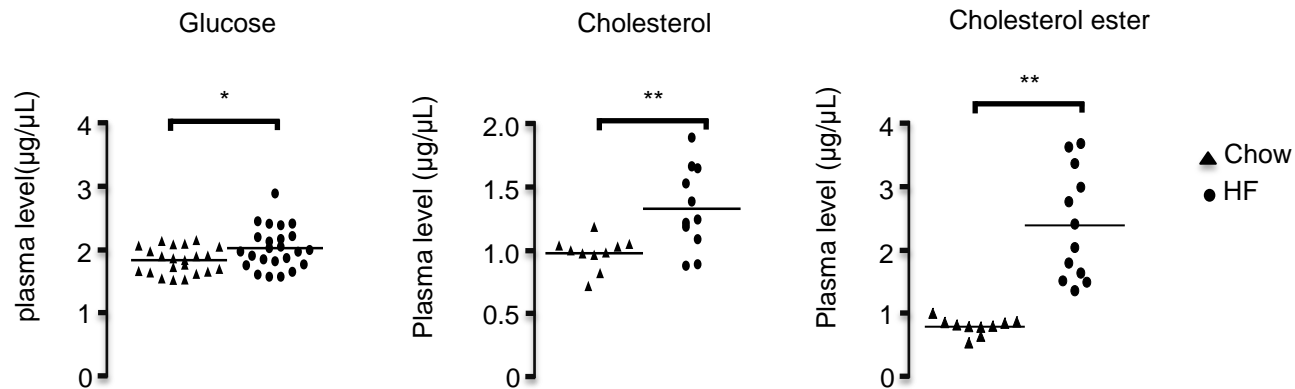

Supplement: S1 Fig — (PDF) [file pone.0139946.s002.pdf]

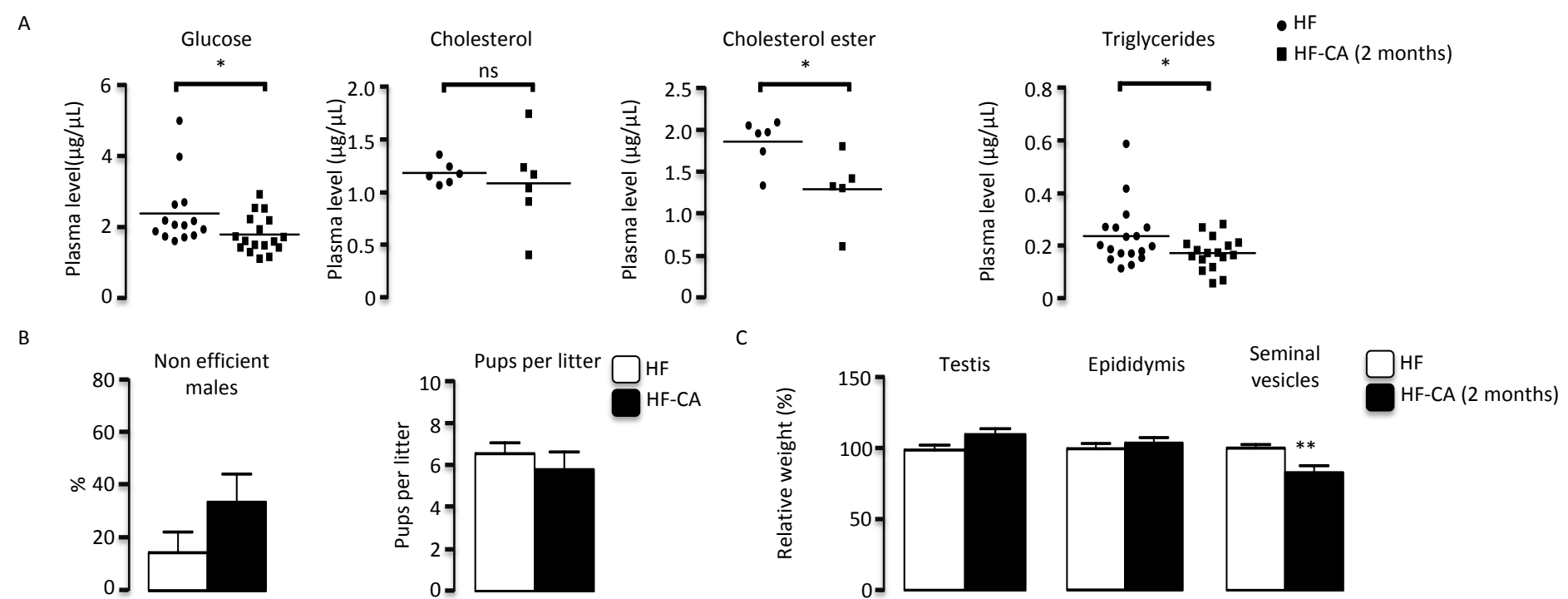

Supplement: S2 Fig — (PDF) [file pone.0139946.s003.pdf]

A

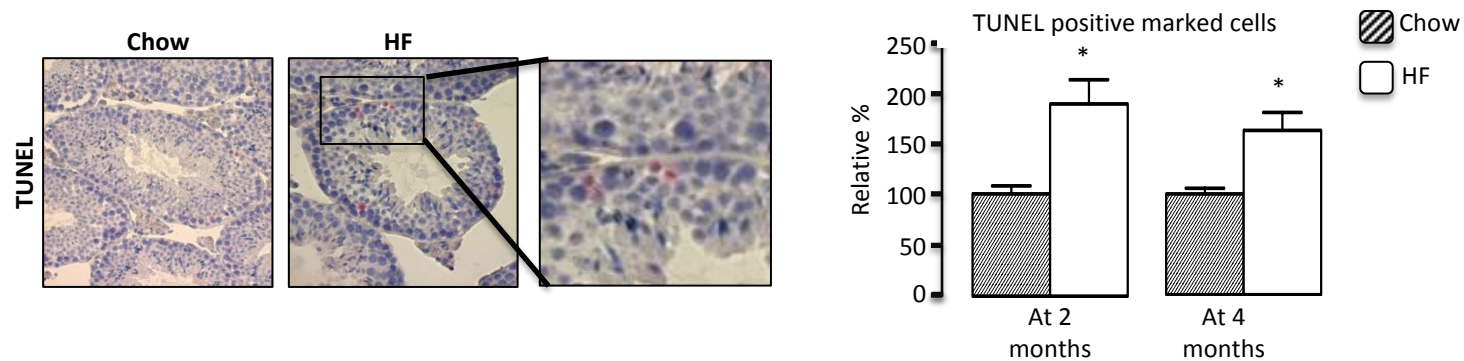

B

**Intratesticular lipid parameters**

2 months

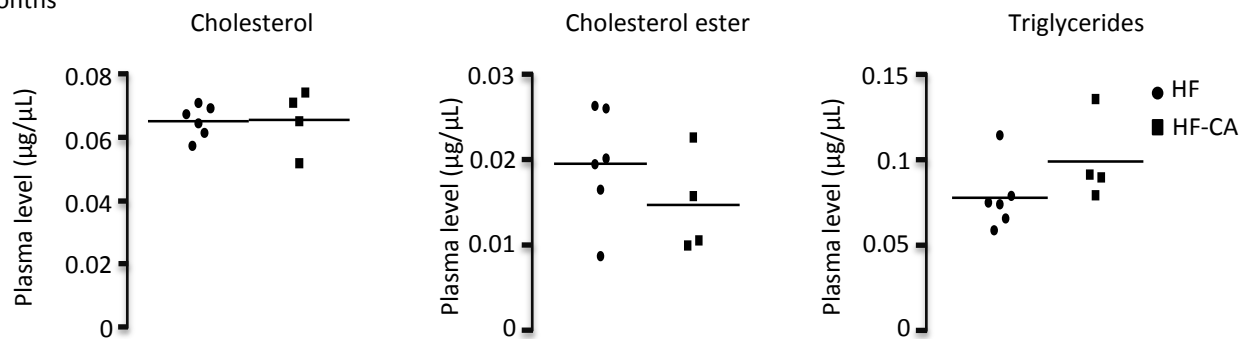

C

**Intratesticular lipid parameters**

4 months

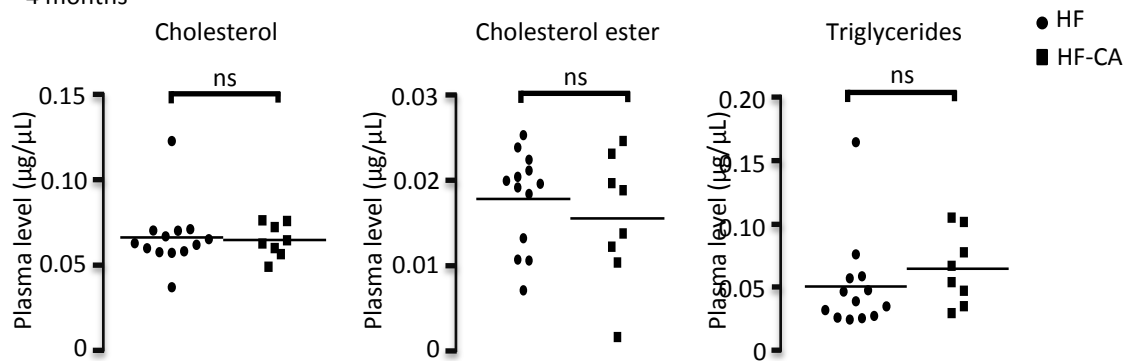

Supplement: S3 Fig — (PDF) [file pone.0139946.s004.pdf]
